# Tightening the purse strings: a stent-free path to a lasting endoscopic gastroenterostomy

**DOI:** 10.1055/a-2318-2829

**Published:** 2024-06-14

**Authors:** Anam Rizvi, Omar Saab, Sanjay M. Salgado, Mohamed Abu-Hammour, Qais M. Dawod, Reem Z. Sharaiha

**Affiliations:** 1 Department of Gastroenterology and Hepatology, NewYork Presbyterian – Weill Cornell Medical Center, New York, United States; 2 Hospital Medicine Department, Cleveland Clinic, Cleveland, United States; 3 Atlantic Medical Group, Summit, United States; 4 Cleveland Clinic Fairview Hospital, Cleveland, United States; 5 Garnet Health Medical Center, Middletown, United States

**Fig. 1:**
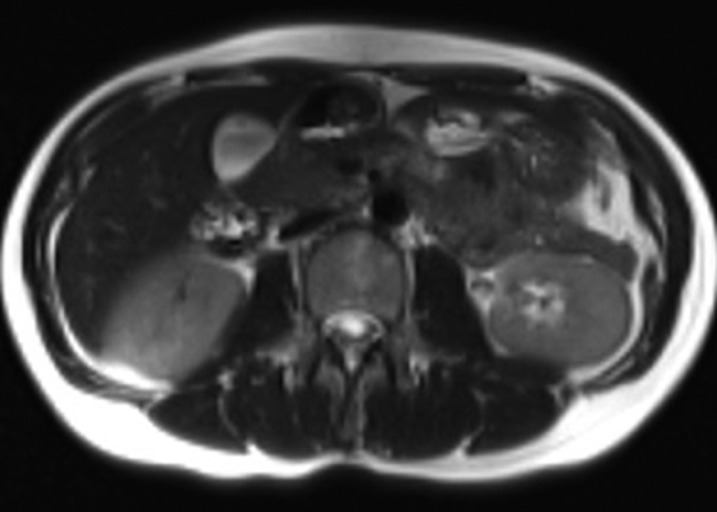
Abdominal magnetic resonance imaging revealed duodenal compression by the superior mesenteric artery, consistent with superior mesenteric artery syndrome.

A 42-year-old woman presented with epigastric abdominal pain for several years. An extensive workup revealed duodenal compression on esophagogastroduodenoscopy (EGD) and superior mesenteric artery syndrome on imaging ( Fig. 1 ). Despite several months of conservative management, the patient remained symptomatic. She deferred surgery and was then offered an endoscopic ultrasound-guided gastroenterostomy (EUS-GE) ( Video 1 ).

Initial endoscopic ultrasound-guided gastroenterostomy, followed by removal of the lumen-apposing metal stent, and endoscopic suturing of the gastroenterostomy anastomosis for creation of a stent-free anastomosis.Video 1

**Fig. 2:**
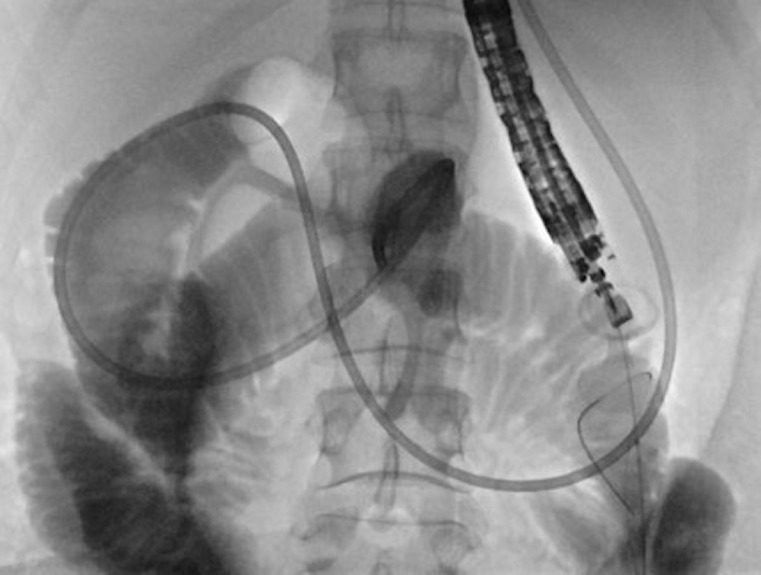
Fluoroscopy image of endoscopic ultrasound-guided creation of a gastroenterostomy with a 15 mm × 10 mm lumen-apposing metal stent.

A successful EUS-GE with placement of a 15 mm × 10 mm lumen-apposing metal stent (LAMS) ( Fig. 2 ) resulted in clinical relief of her symptoms. LAMS was upsized to 20 mm × 10 mm on repeat endoscopy 4 months later.

The patient’s preference was to avoid further stent replacements and therefore the decision was made to suture the gastroenterostomy anastomosis for stent-free patency on repeat endoscopy. Using the OverStitch endoscopic suturing system (Apollo Endosurgery, Austin, Texas, USA), one running suture was placed with eight bites in a purse-string circumferential fashion. To secure the preferred luminal diameter of the gastroenterostomy tract, the suture was cinched around a balloon dilator inflated to 18 mm. Finally, to maintain gastroenterostomy patency as the mucosa healed, a 20 mm × 10 mm LAMS was temporarily placed ( Video 1 ).

**Fig. 3:**
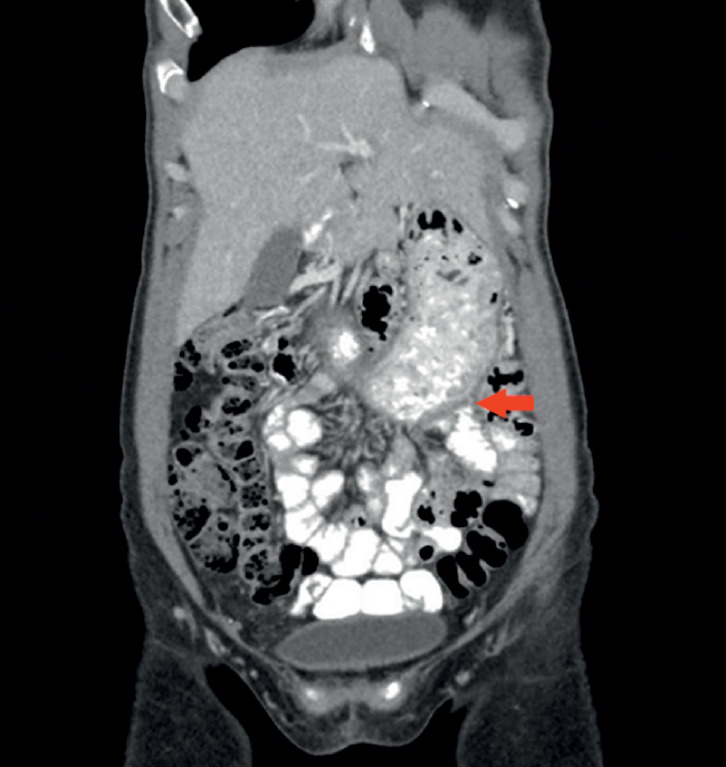
Abdominal computed tomography scan with oral contrast revealed a patent gastroenterostomy anastomosis after removal of the lumen-apposing metal stent.

**Fig. 4:**
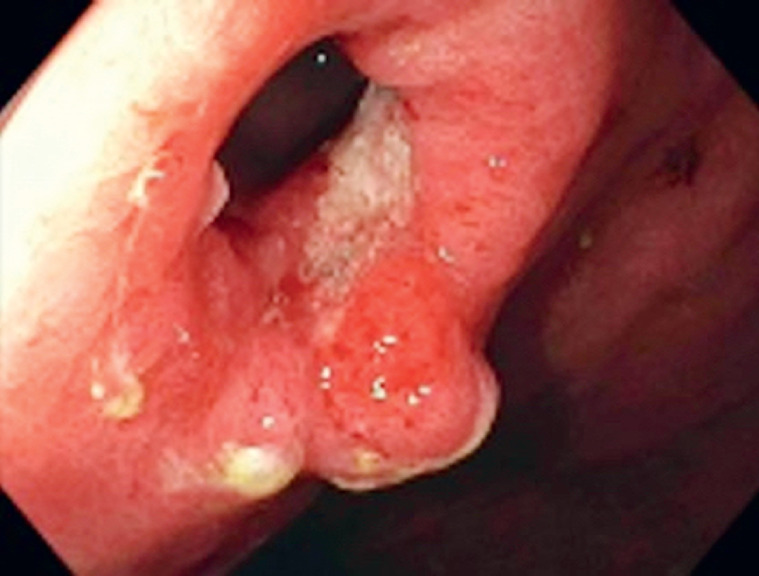
Endoscopy 4 months after stent removal revealed a patent gastroenterostomy anastomosis.

LAMS was removed 3 months later, and the gastroenterostomy was maintained stent-free. Computed tomography scan with oral contrast 2 months after stent removal confirmed patent gastroenterostomy ( Fig. 3 ). A repeat EGD after 4 months affirmed stent-free gastroenterostomy anastomosis patency ( Fig. 4 ). Over 1.5 years of clinical follow-up, the patient remained symptom-free with a patent gastroenterostomy anastomosis.

Our case demonstrates a novel technique to transition an endoscopically created gastroenterostomy to a stent-free approach via suturing the anastomosis in a purse-string fashion. This approach overcomes a current limitation of the technique, typically requiring multiple stent exchanges and in situ stent retention to maintain patency of the anastomosis. Transitioning to a stent-free anastomosis has the potential to reduce complications, decrease healthcare utilization costs, and enhance patients’ quality of life.

Endoscopy_UCTN_Code_TTT_1AS_2AG

